# Formation and Care of the Teeth

**Published:** 1892-08

**Authors:** K. L. Cleaves

**Affiliations:** Montpelier, Vt.


### Formation and Care of the Teeth.

BY DR. K. L. CLEAVES, MONTPELIER, VT.

Read before the Vermont State Dental Society, March, 1892.

I presume all of you are more or less familiar with the subject upon which my paper is written, and perhaps I may not be able to present anything new to you, but possibly it may serve to freshen your memories somewhat on the subjects.

It might well be said of this as of the "fifth pair of nerves," that one has to learn and forget it seven times, before being able to remember it.

When I first commenced the study of the formation of the teeth, I thought it to be an extremely dry subject, but gradually I became more and more interested in it, until now there is no one operation of the whole animal economy in which I am more interested.

Many times a dentist is questioned by the better class of his patients, regarding the formation of the teeth. In such a case it is well for him to have at command a general idea of how and when they are formed, that he may be able to explain to them satisfactorily something about it.

But to go on with the subject, will say that I have abbreviated all that the subject will allow and yet touch on the most imp^rtant points.

Let us first take into consideration the mucous membrane of the mouth. The epithelial layer of the mucous membrane is one and the same thing as that of the skin covering the body. The only point of difference is, the external covering of the body is kept in a dessicated condition by the atmosphere, while the oral mucous membrane is constantly bathed in saliva. Therefore the mucous membrane (of the mouth) and the skin are analagous and continuous structure. In a general way they are composed of two layers—the dermis and the epidermis—yet they have been further sub-divided. At a very early period of fœtal life can be seen the first sign of cellular activity, in the epiblastic layer which covers the gums. By comparing the epithelial covering of the gums, we observe that it has two or more layers of oral cells, while the same membrane on the outer portion of the body has but one. This epithelial covering gradually thickens by the rapid cell-multiplication, until it gives rise to a slight ridge which locates the line to be occupied by the future arch of teeth. Beneath this thickened epithelial layer is a groove, or depression, in the underlying tissue, which gradually deepens, and takes a direction towards the center of the arch, as cell-multiplication advances. This epithelial band (as we will call it) is simply a dipping down of the natural covering of the mouth and is composed of the same elements as the epithelium of the mucous membrane of the mouth. The convex surface of the band is toward the outer side of the jaw. This peculiar curve is almost always seen and is one of the most characteristic features of the band. The walls of the band are composed of the infant layer of cells, while its center is filled with the older cells. The band, as seen at first, is broad, but as it develops and sinks deeper into the jaw it becomes narrower.

The jaw is deepest at the interior portion of the mouth and gradually becomes shallower until it finally disappears in the epithelial covering of the gums at the posterior portion. When this epithelial band is fully formed a lamina is given off from the inner side of the band. The formation of this lamina may be explained as W-shaped enfolding of the infant layer.

These two processes are termed the band and lamina. This lamina is only a continuous process of the epithelial band. Cell-multiplication seems to come to a stand in the band, while it still proceeds in the lamina, dipping deeper into the substance of the jaw. At this period the band becomes shallower and sometimes disappears, while the process of development is transferred to the lamina. Small buds make their appearance on the free margin lamina, and soon extend into slender cords, each cord forming in time the enamel organ of the temporary tooth. These buds correspond in number and position to the future deciduous teeth. The length of the cord varies, being shorter in the human than in some of the other mammals, and is also shorter in the temporary set, as those of the permanent have to descend beyond and beneath them.

The cord is composed of a solid ingrowth of cells which constitute the lamina from which it arises. These cells are oval or spherical in shape. They are sometimes spoken of as cylindrical in shape, when the layer is only one cell deep, but if more than one layer exists I believe they are usually spoken of as being oval or spherical. The cord at first pursues a horizontal course, until it attains considerable increase in length, when it turns sharply upon itself and takes a vertical direction more or less deeply into the substance of the jaw. After the cord has changed its direction, its extremity assumes a bulb-shaped mass. The part of the cord which forms the connecting link between the bulbous portion and the band, does not keep up with the extremity in growth, therefore it is rightly named the neck of the enamel organ.

At this stage of the development of the enamel organ the dentinal germ or papilla makes its appearance, which causes the enamel organ to become concave, although it does not become separated from the sub-epithelial connecting tissue of the jaw, and its growth is upward, while the growth of the cord has constantly been dipping downward.

Should we examine the enamel organ at about the fifteenth week of the development of the embryo, we would find that a considerable change had taken place.

The primitive elements (composed of polygonal cells in the centre with an external layer of prismatic cells) have been notably modified. The cells of the central mass have been notably modified. The cells of the central mass have been changed into the stellate cells. These stellate cells are composed of a central nucleus, surrounded by a finely granular mass, which inosculates with the adjoining elements. The cells near the periphery maintain their primitive polygonal form for a time, but as the enamel organ increases in size they become stellate. After a time the base of these stellate cells presents the regular prismatic form of a hexagon. During the early stage of evolution the prismatic cells have the same characteristics on all parts of the periphery, but as soon as the papilla make their appearance those of the external or convex surface diminish in size until they finally disappear, while those of the internal or concave surface remain for the formation of the enamel organ. The cells on the concave surface of the enamel organ increase in length and their extremities form the slender process which are continuous with the filaments of the surrounding cells and constitute what is known as the stratum intermedium. This stratum intermedium consists of cells which have not become stellate, or we might call them young stellate cells. The enamel forming cells are developed from embryonal corpuscles, and these corpuscles are formed by the breaking or dividing of the prismatic cells. The cuticula clentis or "Nasmythe membrane" is formed from the enamel cells and may begin to form before any calcification of the dental tissue beneath. It may be seen on the surface of the enamel, after the eruption of the crown of the tooth, and may be separated from the surface of the enamel by the use of strong acids.

As the epithelium is undergoing this peculiar and interesting process of development into the enamel organ, we find a projection of the corium of the mucous membrane rising up from the dental groove to meet it. This is called the dental papillæ, which papillæ are to form the bulk of the teeth. The first germ of the dentine appears as a dark semi-lunar mass at the bottom of the dental groove. At certain points where the subsequent teeth are to be, this young structure developes and pushes up against the enamel germs.

The papillæ is made of connecting tissue cells, similar to those found in other parts of the body. As the dentinal papillæ increases in size and pushes up against the enamel organ, it becomes constricted at its base, about where the enamel organ is reflected back upon itself.

The follicular wall [which begins to develop soon after the dentinal germ] gradually grows upward and embraces the enamel organ and papillæ, soon assuming the appearance of a distinct laminated membrane, which may be separated from the adjacent tissue except at the base of the papillæ. The dental follicle gradually grows upward until it gradually closes over the top of the enamel organ, beneath which is the papillæ, and the coming together of its edges produces strangulation of the epithelial cord, therefore the cord becomes ruptured at this point. The dental follicle also becomes separated from the mucous membrane.

At this stage the cells from the dentinal papillæ form a soft mass of animal material, which afterwards become filled in with lime salts to complete the dentinal tissue. After the dentinal papillæ has become covered over by the enamel organ, odontoblasts [dentine cells] begin to form.

The odontoblasts belong to the connective-tissue group, and are formed from the dentinal papillæ. The odontoblasts are seen as elongated cells, with numerous fine processes extending into the calcareous mass. As the processes, which the odontoblasts send out develop, they calcify on the outside, forming the dentine, while the uncalcified portion forms the dentinal fibrillæ. We might say that the odontoblasts superintend the depositing of the lime salts around the rod-like febrils, and thus tubular dentine is formed. The deposits of dentine are the work of older cells on the surface of the dentinal pulp. Dentine is formed by the secretion of the lime salts around the fibrils of the odontoblasts, and these being rod-like, it is very plainly seen how the deposition takes a tubular form. The thickening of the dentine is by accretion of lime salts in such a way as to lengthen the tubuli. These cells persist throughout the life of the pulp, and have the power to form secondary dentine, when they are stimulated by the irritation of decay. This thickening is at the expense of the pulp cavity and of the size of the organ itself.

The remains of the odontoblasts is that layer which constitutes the investment of the pulp, lying between the nerves and vessels and the dentine.

Let us say a few words in regard to the development of the cementum. This process is only slightly modified from that of the development of bone. The circumference of the root before the deposition of cement is as large as it ever becomes, as the increase in thickness of the dentine is from the periphery toward the center at the expense of the size of the pulp. The thickening of the cement is from the first layer deposited upon the dentine externally, thus enlarging the circumference of the root. When this process extends beyond a certain limit, we have what is called exostosis, generally occurring later in years, from the result of constant irritation. The pericementum is very much like the periosteum, and superintends the deposition of the cementum. A single layer of osteoblasts or cementoblasts is first found around the outside of the dentine. Other layers follow, each assuming the characteristics of the first formed layer, until finally the desired thickness is reached.

We have seen how beautifully the process of development of three of the physiological divisions of the teeth is performed by the economy. Now let us direct our attention to the fourth, which is the dental pulp. The pulp occupies the pulp cavity in the center of the tooth.

We find the pulp to be the remains of the dentinal papillæ, after it has performed its work of dentinification. We also find that the dentine receives its nerve and blood supply through the pulp. The nerves of the pulp are many, and consist of medulated and non-medulated fibers, which enter the pulp through the apical foramen. The pulp is an extremely sensitive organ, and is enclosed in a very delicate, almost structureless membrane, which is attached to the walls of the pulp cavity. A great many theories have been advanced in regard to the termination of the nerves, and I believe no definite knowledge has yet been presented. It is claimed by some that the fibers pass between the odontoblasts and either unite with the dentinal fibrils or pass between them into the dentinal tubuli. Others assert that the nerves become united with the stellate cells, which form a layer beneath the odontoblasts. The dental pulp, as age advances, diminishes considerably in size. Sometimes the degeneration is carried on to such an extent that the pulp becomes a shriveled insensitive mass.

What has been said so far has reference to the deciduous teeth. The permanent teeth, which later on are to take the place of these, arise from an epithelian cord, which originates from the cord of the corresponding deciduous tooth. This, however, does not hold good for all of the teeth, only, for instance, the incisors, cuspids and bicuspids. The permanent cord takes a vertical direction when sinking into the substance of the jaw, and at the same time assumes a spiral form. It can always be distinguished from the primitive cord by this peculiar spirality. This spirality of the permanent cord is necessary, as it permits the follicle to reach a point below a temporary tooth without stretching the cord. The origin of the cord for the permanent teeth is not always at the same time, but varies considerably.

In the human embryo, the cord for the permanent incisors may be first seen about the fifth month, while we find that their eruption does not take place until between the years of six and eight. We find that the permanent molars, which have no deciduous predecessors, originate in a different manner from those which have deciduous predecessors.

The follicle of the first permanent molar appears only a short time after the appearance of three of the deciduous teeth, which is about the fifteenth week of embryonal life, and does not erupt until about the sixth year, therefore it is called the sixth year molar. It originates directly from the epithelium of the mucous membrane, back of the temporary teeth, where no follicle has preceded it.

The second permanent molar originates from the epithelial cord of the follicle of the first permanent molar, in the same manner as do the twenty anterior teeth. It takes a position back of the first permanent molar and is developed in line with those anterior to it. The origin of the third permanent molar or wisdom tooth, takes place in the same manner as that of the second permanent molar. Although we have said that the cords of the twenty anterior permanent teeth arise from the follicles of the temporary, yet sometimes they emanate directly from the epithelium of the mucous membrane. After the epithelial cord becomes detached from the enamel organ, it generally disappears by absorption, yet it is possible for some such masses to be left and become the enamel organs of supernumerary teeth.

Now, in conclusion, will say something in regard to the care of the teeth, as a suggestion in regard to what we dentists, as a rule, fail to do, and that is to impress upon parents the importance of proper food materials while the teeth are forming.

It is too often the case that the first visit of a child to a dentist is after the first teeth have come and gone, many of them, and the permanent teeth begin to give them trouble. Their deciduous teeth being extracted by their parents with a string, or they work at them themselves, until they can pick them out with their fingers.

About the time the six year molar is too badly decayed to save, without destroying its nerve, and after being made to believe from infancy that the dentist is the most horrible of horrible creatures, the child is presented with the question: "Doctor, why is it that children's teeth don't last as they used to?" Now this is exaggerated in many cases, but none of you can deny that it's truth has been too often verified in your own experience.

Now for seven months before until seven months after birth the first deciduous tooth is growing atom by atom as the necessary elements of tooth substance are furnished by the mother's blood. And at the time of birth the germs of the permanent teeth are hidden in the gums, gradually forming, not to be called into active service until many years later.

Now, if the deciduous teeth are of so much importance as to require fourteen months for growth and development, while nine is sufficient for the eye and ear, I think they are of too much importance to suffer through neglect and carelessness. Commencing as far back as fœtal life, when the child receives its nourishment from the mother's blood, can you introduce the proper food elements which will lead to a more perfect set of teeth?

The child, while dependent upon its mother, gets lime, phosphorous, potash and all the other elements of which the teeth are composed, just in proportion as the mother gets them. If the mother lives principally on starch, butter and sugar, neither of which contains a particle of tooth-forming material; would it be strange if the child had poor teeth, especially teeth poorly enameled. Now, I think there are well defined principles, which, if closely carried out during the formation and growth of the teeth, would partially if not wholly overcome this difficulty. In talking with one man quite recently on this subject, he said: "Why, if teeth should be brought up to that standard, where they needed no filling at all, what would you dentists do for a living?" I hardly anticipate such a time when there will not be plenty of teeth to fill. But I believe there is a method (even in our generation) by which teeth can be improved to such an extent, that when a dentist exercises his utmost skill and care in filling a cavity, he will not do so with the feeling that, perhaps, in a year or two it will come back to him decayed around the filling.

Dentistry at the present time has reached that standard, where it stands shoulder to shoulder with the medical profession. In the colleges anatomy and physiology, and many other branches are taught as thoroughly as at the medical schools. Not enough attention is paid to those branches in dentistry. The manipulative part in dentistry has already reached a high degree of excellency. When a conscientious dentist, who thoroughly understands his business, fills every tooth the best he knows how, with the material he thinks is best adapted for that tooth, he is doing all that can be done in that direction. Then, when teeth fail under such proficiency, as they sometimes do, what is to be done?

Patients should comprehend that the finest and best work will not preserve teeth in defiance of their abuse and carelessness. My opinion is that every dentist should impress upon his patients these facts:

Instruct mothers in regard to the best articles of food for their growing children.

Instruct parents as to the means of preventing the premature loss of the deciduous teeth and preservation of the permanent ones. And too, the importance of thorough cleanliness at all times. In regard to articles of diet, it would be well for us to familiarize ourselves with the constituents of tooth substance and where they may be found.

We would be safe in telling our patients that everything that grows will make good teeth, if eaten in its natural state, no elements being taken out. Horses, cows, sheep and other animals that live on nature's own production have good teeth. For children to be strong and healthy and have sound teeth, it would hardly be necessary for them to eat grass or go back to a state of savagery, but take food in the proper proportion as nature provides it. For instance, phosphate of calcium is the principal element in tooth substance. That is found in milk, eggs, potatoes and many other vegetables and fruits, but especially in the grains or cereals. It is most abundantly found in wheat " the staff of life," but not in the fine white flour, that the good housewife is so fond of making into snowy loaves of bread. I would advise the free use of lime water with the milk, as much as would be assimilated not to be distasteful. Would also advise it as a mouth wash after the use of acid fruits, lemonade, etc. It is very easily prepared and inexpensive. A teaspoonful in a glass of water or milk would not be noticed and would have a wonderful tendency towards hardening the teeth. Even as a mouth wash a portion of it would be taken up by the absorbents and eventually reach the teeth through the general circulation. I

will not further discuss the articles of food, but will leave it to your better minds to consider.

I hope the time will come when people will pay more attention to diets containing tooth-forming material and arrive at results that many already have attained.

